# Management of pain for people with dementia living in nursing homes: stakeholders' perspectives on a prototype of a complex intervention

**DOI:** 10.3389/fpain.2026.1868719

**Published:** 2026-08-19

**Authors:** Caroline Kreppen Overen, Adelheid Hummelvoll Hillestad, Maria Larsson, Cecilia Olsson, Siren Eriksen

**Affiliations:** 1 Faculty of Health, Science and Technology, Department of Health Sciences, Karlstad University, Karlstad, Sweden; 2 Lovisenberg Diaconal University College, Oslo, Norway

**Keywords:** complex intervention, dementia, nursing, nursing home, pain assessment, pain management, palliative care

## Abstract

**Background:**

Healthcare personnel often report uncertainty about how to identify and alleviate pain in people with dementia. Up to 80% of nursing home residents with dementia are estimated to experience chronic pain. There is a lack of structured pain management interventions that focus on integrating and building upon the individual resources of people with dementia in the nursing home context. Although a prototype of such an intervention has been developed, further knowledge is needed regarding its functioning in clinical practice, relevance, and capacity to meet clinical needs. Stakeholders possess substantial field-specific knowledge. In this study, the prototype of the intervention is presented to stakeholders to increase the likelihood of a clinically relevant intervention and successful implementation.

**Objectives:**

To describe stakeholders' perspectives of the prototype regarding intervention content and relevance, and implementation strategy.

**Methods:**

Seven dyadic interviews were conducted with practice development nurses and first-line managers in nursing homes between May to June 2025 to describe their perspectives on the prototype. The transcripts were analyzed with a deductive framework analysis.

**Results:**

The intervention was regarded as relevant and as having the potential to enhance healthcare personnel competence, structure pain management, and integrate residents' resources. However, successful implementation requires integration into existing practice models, and potential moderators were identified at both the organizational and individual levels.

**Conclusions:**

This study presents an applied approach to collaborating with stakeholders in intervention development. The participants’ perspectives on activities, moderators and potential outcomes of the intervention provides essential knowledge for further refinement of the intervention and future evaluation.

## Introduction

Pain is common and often persistent among nursing home residents with dementia. Reported prevalence varies across studies and measurement approaches, but up to 80% are reported to experience pain (1). Untreated pain can have severe consequences, including reduced functional ability (2, 3), depression (4) and diminished quality of life (5). Pain management for people with dementia is internationally recognized as a significant challenge for healthcare personnel (HCP), who often report uncertainty about how to identify and alleviate pain (6). Over time, many individuals with dementia lose the ability to verbally express pain and contextualize their pain experience (7, 8). As a result, pain may manifest through behavioral and psychological symptoms such as aggression, apathy, agitation, or wandering (9, 10).

In this study, pain management is understood as healthcare personnel's (HCP) clinical practice related to a resident's pain and/or potential pain, grounded in the Theory of Symptom Management (TSM) (11, 12). TSM is a middle-range nursing theory that provides a framework for understanding the relationship between symptom experience, symptom management strategies and symptom-related outcomes. TSM underscores that recognition and assessment of symptoms constitute foundational steps in symptom management, because these processes shape all subsequent clinical decisions. TSM emphasizes individual variability and the need to tailor symptom assessment and treatment to each person's unique symptom experience and context. The context is conceptualized through the interplay between person, environment and health and illness characteristics (12). In the case of pain in people with dementia, the health and illness dimension, including progressive cognitive impairment and communication difficulties, the person with dementia, including remaining verbal abilities, biography and individual patterns of non-verbal embodied communication, and the environmental dimension, including the nursing home setting, staffing, routines and culture, jointly influence how pain is expressed, perceived and acted upon (12). As pain is a subjective experience, remaining verbal abilities and individual non-verbal embodied communication are therefore important resources in pain management for people with dementia (13). The progression of dementia occurs gradually, and within nursing homes each resident will have different remaining abilities and individual resources that are central for pain recognition and assessment in pain management (14). People with moderate to severe dementia are heavily reliant on others to interpret, respond to, and communicate their expressions of pain (14, 15). In line with TSM, decision-making in relation to pain management in this context has been described as highly complex, and the importance of pragmatic and flexible approaches has been highlighted (16).

A hierarchy of pain assessment is recommended to guide pain assessment, where several complementary elements can be combined to determine the presence of pain: (a) awareness of potential sources of pain, (b) attempts to obtain self-report, (c) observation of patient behaviors, (d) proxy reporting of pain and changes in behavior, and (e) an analgesic trial (15, 17). In addition, regular assessment, post-intervention reassessment, and systematic documentation are recommended (15, 17). However, emerging research indicates a persistent gap between these evidence-based guidelines and everyday clinical practice. For instance, Resnick et al. (18) show in their study that HCP do not adhere to these recommended practices. Similarly, in a recent review, Li et al. (19) highlight a substantial lack of knowledge and suboptimal practice among HCPs regarding pain management for people with dementia. They emphasize that critical gaps remain in addressing the specific challenges of pain management in this population, and that only a small minority of HCPs follow established standards or use validated pain assessment tools in routine practice. The use of observational tools and observation skills to identify pain can help compensate for lack of verbal communication (17, 20, 21). Several pain assessment tools for people with dementia have been developed and evaluated (22, 23), and the systematic use of standardized observational tools is recommended (23). However, such tools only capture parts of the overall picture and must be interpreted in context (12). Quantitative pain measures are important, but often miss key aspects of the subjective experience, such as personal context and meaning, which significantly influence how pain is experienced (24). Pain management models that address this subjective dimension are therefore essential (24). In their meta-analysis, Tsai et al. (25) investigated the effectiveness of interventions to improve pain assessment and management in people living with dementia, concluding that comprehensive and multifactorial pain models improve nurses' pain management compared to the implementation of standalone assessment tools. However, none of the included interventions emphasize a structured approach to harness individuals' residual capacity and resources. There is a notable lack of structured pain management interventions that focus on integrating and building upon the individual resources of people with dementia in the nursing home context.

This study is part of the development of a complex intervention targeting HCP pain management for people with dementia living in nursing homes, included in the project Palliative care to Older People with dementia, with the overall purpose to develop and test models for palliative nursing in this target group. The intervention development aligns with the Medical Research Council (MRC) framework for developing and implementing complex interventions, situated in the development phase (26). The MRC Framework defines complex interventions as those containing several interacting components, where complexity may arise from multiple potential outcomes, diversity in target population or the adaptability of implementation methods (26). This intervention aimed at pain management for people with dementia in nursing homes is considered complex, as it involves multiple actors and processes, and a variable and often unstable target population and context. This underscores the importance of planning implementation and context-sensitive facilitation strategies from the outset, before formal evaluation and large-scale implementation (26).

Our three prior studies focused on identifying the current state of evidence, determining the needs of both recipients and providers, understanding existing practices and context, and developing the theoretical foundation for the intervention (14, 27, 28). Pain management processes in nursing homes can be understood as a process that integrates HCPs clinical practice with the pain expressions of people with dementia. First, *pain awareness* refers to being alert to and actively searching for pain, and relates to HCPs' recognition of potential pain, their alertness, knowledge, and understanding of the situation. This depends on continuous observation and assessment of residents' body language and reactions in daily life (27). Second, *suspected pain* arises when HCPs identify potential pain based on four key indicators: behavioral changes, signs of pain, verbal self-report, or known reasons for pain (27). Recognizing deviations from a resident's usual state is crucial, and these changes are often subtle and require both sensitivity and familiarity with the residents (14, 27, 28). Third, *pain mapping* involves further investigating of this suspicion, aiming to identify the underlying cause of the pain expression. This include talking with the resident, using assessment tools, reviewing medical records, conducting physical examination, and drawing on perspectives of other HCP and/or significant others (14, 28). Measures ideally target an identified underlying cause of a pain expression, but in clinical practice the cause is often uncertain (14, 27, 28). “Trial and error” are therefore described as a common strategy, where different combinations of pharmacological and non-pharmacological interventions are tested to address potential cause, with the goal of restoring baseline functioning and reducing or eliminating behavioral symptoms (27). Finally, evaluation of measures focuses both on whether the initial reason for intervention has been addressed (14, 27, 28) and on identifying any unintended consequences of the actions taken (14, 28). Pain management is a continuous process, both in the presence and absence of pain expressions (14, 27).

Further, the results stress the need for structured assessment processes to optimize pain management and a crucial importance of appropriate professional competence in pain management for people with dementia (28). Based on the findings from the three prior studies, a prototype of the intervention was developed. The intervention targets how HCP in nursing homes can systematically recognize, assess and evaluate pain in people with dementia. The primary focus of the intervention is to support people with dementia in using their resources and individual abilities to express, describe, or self-report pain, and integrate these expressions into a comprehensive pain management process.

A core element of the MRC framework is to specify the intervention's key components, the mechanisms through which it is expected to work, the intended outcomes, and the potential influence of contextual factors (26). Through the development of the prototype, we had an initial idea of how the intervention would work. However, there was a need for more in-depth knowledge about how the intervention can function in clinical practice, and whether it addresses the needs of clinical practice. Stakeholders possess substantial field-specific knowledge, and their insights are pivotal to develop an intervention relevant to clinical practice and more likely to achieve successful implementation. Presenting the proposed intervention to stakeholders prior to testing provides an opportunity to evaluate its potential relevance and utility, identify potential moderators, and assess whether contextual needs differ across nursing home units. This approach can offer valuable insights into the level of flexibility required for the intervention to achieve its intended outcomes and to support successful implementation over time (29). Thus, to increase the likelihood of a clinically relevant intervention and successful implementation, we presented the prototype of the intervention to stakeholders, with the aim of describing stakeholders' perspectives of the prototype regarding intervention content and relevance, and implementation strategy.

## Methods

We employed a descriptive design utilizing dyadic interviews (30). Dyadic interviews facilitated both interaction and in-depth discussion, enabling the integration of two distinct perspectives while ensuring ample speaking time for both participants. This manuscript is written in accordance with the SRQR checklist (31).

### The intervention content

**Figure 1:**
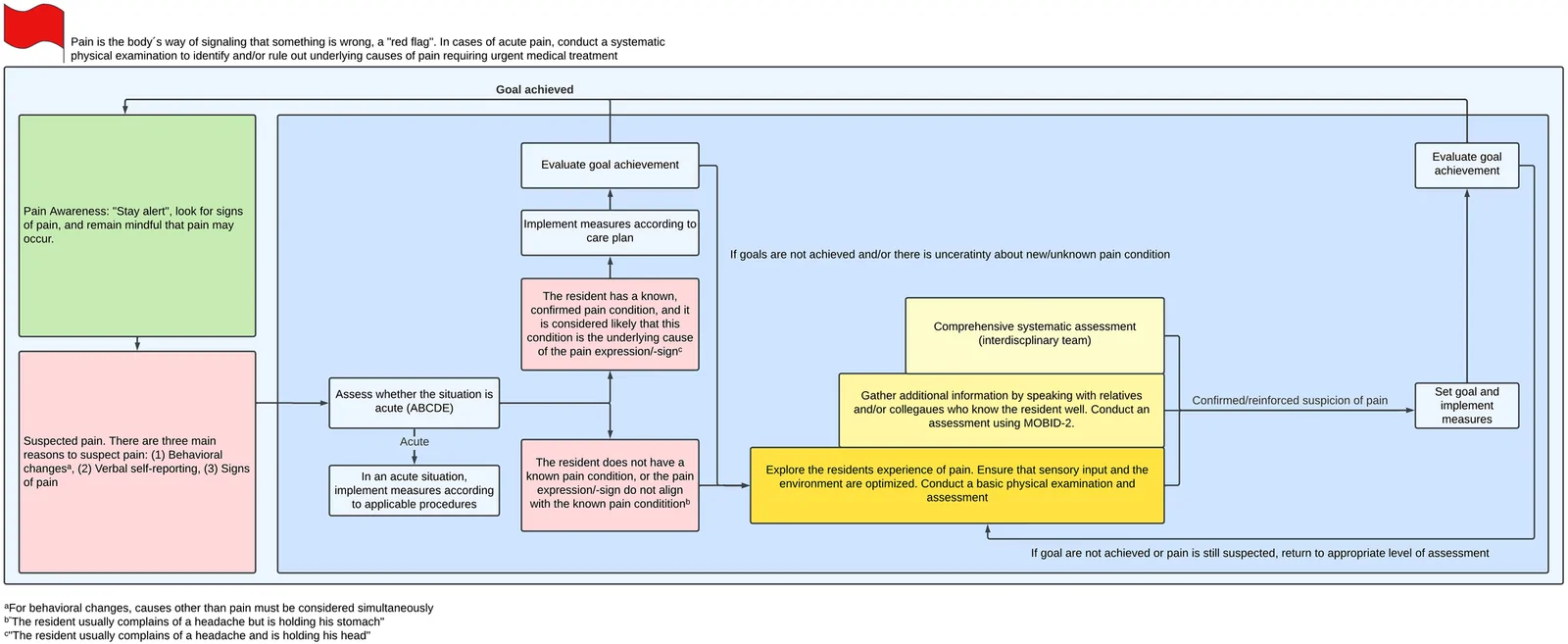
Flowchart.

The content of the proposed intervention is twofold and consists of (a) educational modules and (b) a flowchart (Figure 1) structuring the workflow for HCP. The educational modules (Table 1) provide the knowledge necessary for appropriate processes in accordance with the flowchart. The flowchart is accompanied by written information that explains its purpose and structure. The flowchart illustrates the distinct phases of pain management in people with dementia: pain awareness, suspected pain, and the mapping phase. The mapping phase represents a systematic approach to identify the underlying cause of pain expressions, implementing measures, and conducting evaluations. The content aligns with the principles of the pain assessment hierarchy (17), and is grounded in TSM (12).

**Table 1:** Educational modules.

Module	Content
1: Pain	Types of pain Perspectives on pain
2: Dementia and pain expressions	How dementia affects pain reporting and the approach it requires Resources of people with dementia in pain management: how people with dementia express pain (including behavioral and psychological symptoms of dementia) Person-centered pain management: the importance of knowing the person
3: Communication with people with dementia	How to support people with dementia in self-reporting: communication techniques, optimizing sensory input and the environment Behavior as communication
4: Recognise, assess and evaluate pain	Methods for uncovering underlying physical causes of pain, including basic examination techniques (videos/opportunities for practical training) Trial and error—testing interventions as part of the assessment process Use of assessment tools MOBID-2, behavior chart The different levels of assessment (flowchart)
5: Pain management processes in a system	Appropriate and systematic evaluation of measures Documentation structure: writing effective pain reports Flowchart: the process

The proposed content of the intervention has been reviewed and discussed by an expert group to help safeguard the quality of the content. The group consisted of three RNs (two professors in nursing and one assistant professor) and one physician (professor) from various fields related to pain, dementia, and nursing home care.

### The intervention context

The intervention prototype is developed in a Norwegian context. The national recommendations currently available for pain management for people with dementia recommend the use of assessment tools. However, there is a lack of pain management nursing procedures or care models that frame the use of assessment tools for the target group. In Norwegian nursing homes, three main categories of nursing staff are employed: Registered Nurses (RNs), Certified Nursing Assistants (CNAs), and Nursing Assistants (NAs). RNs and CNAs are licensed healthcare professionals, with RNs required to hold a bachelor's degree. In contrast, no formal health education is required for NAs (32). NAs frequently work additional shifts and have diverse educational backgrounds ranging from no formal qualifications or non-health-related training to current nursing students. NAs, CNAs and RNs collaborate with physicians, and sometimes other professionals such as physiotherapists and occupational therapists, in the care of people with dementia. Norwegian nursing homes, with their diverse actors, including residents, significant others, staff, local and political leadership, function as a complex system (33). They frequently adapt to unpredictable internal and external factors, such as staffing level, staff skills and attitudes, and residents' needs (33), all of which can influence clinical pain management and the implementation of pain management interventions.

Many Norwegian nursing homes employ practice development nurses, whose responsibilities include quality improvement and staff development, often combined with direct resident care. First-line managers are also key to development efforts in nursing homes (34). Most first-line managers are RNs, with responsibilities spanning personnel management, financial oversight, and resident care (35). Together, practice development nurses and first-line managers possess a combination of patient- and staff-related expertise, as well as organizational and systemic knowledge, making their competencies particularly relevant to the aim of this study. This dual perspective was considered important when selecting stakeholders, as it aligns with the need to account for context in intervention development and implementation (26).

### Study specific context and participants

The 14 participants in this study represented seven units from four nursing homes in urban and suburban areas of southeastern Norway. Each of the seven dyads represented one unit and consisted of one practice development nurse and one first-line manager. Each of the seven dyads constituted a single unit specializing in dementia care; five were special care units and two were enhanced special care units. The nursing homes had an average of 76 beds (range: 42-127), and the included units had an average of 15 residents (range: 10-17). The participants comprised seven practice development nurses and seven first-line managers, all of whom were RNs. One practice development nurse was organized across units. Demographic data are presented in Table 2.

**Table 2:** Demographic data of the included RNs.

Demographics	First-line managers (*n* = 7)	Practice development nurses (*n* = 7)
Gender	7 women	7 women
Age	Range 36–51 years (median = 44)	Range 31–56 years (median = 38)
Relevant ECTS credit courses^a^ after bachelor's degree	Total (*n* = 6); leadership (*n* = 5)	Total (*n* = 6)
Length of time working with people with dementia	Range 12–31 years (median = 26)	Range 4–33 years (median = 10)
Length of time working at the specific unit	Range 2–13 years (median = 7)	^b^Range 1 month—8 years (median = 3,75)

a Including palliative care, dementia care, advanced clinical nursing, mental health, leadership, pedagogy and supervision.

b One participant was organized across units and did not apply this data.

Participants from two of the nursing homes were strategically recruited based on their roles within the units, through an established research collaboration and by contacting the nursing home managers at each facility, who provided information about the study to potential participants. Participants from the two remaining nursing homes were recruited via snowballing (36). Participants received written and oral information about the aim of the study, the voluntary nature of participation and written informed consent to participate was obtained.

### Data collection

Seven dyadic interviews (30) were conducted during May to June 2025. One week in advance, a description of the intervention and a semi-structured interview guide were sent to the participants. The interviews took place in a meeting room at the participants' workplace, lasted approximately one hour, and were recorded using GDPR-compliant software for automated transcription. The interview guide is presented in Table 3.

**Table 3:** Interview guide.

Interview focus	Questions asked
Content of the intervention: perceived relevance and utility, adaption and suggestions for improvement	- ^a^Based on the educational modules: Is the content relevant? Why/why not? Are there any changes that should be made? - ^a^Based on the flowchart: Is the content relevant? Why/why not? Are there any changes that should be made? - Pain management involves multiple professional groups: Who does what, and what responsibilities do the different roles have?
Implementation of the intervention: Strategy and expected feasibility	- How can this intervention be implemented in your nursing home? - How do you think it would be to implement this intervention in your daily work? What factors could facilitate or hinder its implementation? - ^a^How can the implementation be evaluated? - How can we best ensure that the intervention is sustained over time? What factors might hinder its continued use in the long term?

a When addressing questions of relevance and evaluation, the participants discussed potential outcomes.

Providing materials in advance of the interview required the researcher to address participants' questions during the interview, as they sought clarification on the material provided. This necessitated that the researcher carefully balanced maintaining sufficient proximity to answer the questions and guide the interview productively, while also ensuring sufficient distance to avoid overly influencing the interview situation (37). The adequacy of the data to capture similarities, nuances, and differences in the material was continuously assessed, and it was deemed appropriate to stop data collection after seven interviews. At that point, the dataset was considered sufficient to conduct the planned analysis.

### Data analysis

Inspired by Mills et al. (38), we conducted a deductive framework analysis, categorizing participants’ perspectives into the core constructs of an intervention: activities, moderators, and outcomes. *Activities* refer to the intervention content (educational modules and flowchart) and the implementation strategy, which activate mechanisms, such as HCP working according to the flowchart. *Moderators* are factors that influence these mechanisms, shaping how and to what extent the intervention works, either positively or negatively. Activated mechanisms lead to change, resulting in *outcomes* (38). A matrix approach was used to identify similarities and differences across dyads, with each dyad analyzed as a single entity.

We followed the framework analysis steps by Gale et al. (39). Given that our framework was predefined, we adopted the following steps: Transcription; Familiarization with the interview; Applying the analytical framework; Charting data into the framework matrix and; Interpreting the data. Using NVivo (40), data segments from the interview transcripts were coded according to the predefined framework and assigned thematic codes. Subsequently, the coded meaning units were condensed into descriptive, summarized units and transferred to a matrix spanning all dyads to visualize similarities and differences between the dyads. The first author (CKO) applied the framework to two interviews and presented the initial matrix to the research group for discussion. As the applied framework effectively captured the data, with minimal information falling outside its scope, we proceeded with this approach. The matrix, including descriptive data segments covering all dyads, was reviewed by the whole team before the write-up.

### Ethical considerations

This study was approved by the Norwegian Regional Committees for Medical and Health Research Ethics (459187), Norwegian Agency for Shared Services in Education and Research (615148), and the Ethics Committee at Karlstad University (Dnr. HNT 2025/293). Ethical reflection and considerations, in accordance with the ethical guidelines in the Nordic countries (41) and the Declaration of Helsinki Ethical Principles for Medical Research (42) were integrated throughout the entire study period. Data were securely collected and stored according to guidelines at Karlstad University and Lovisenberg Diaconal University College.

## Results

The results are organized and presented according to activities, potential moderators, and potential outcomes.

### Activities

Activities refer to the content of the intervention, including the implementation strategy. The dyads' perspectives on activities are presented in two sections: intervention components (flowchart and educational modules) and intervention strategies. The dyads expressed a significant need for the intervention, emphasizing its high relevance.

#### Intervention components (educational modules and flowchart)

All dyads highlighted the importance of appropriate knowledge and competency levels among different groups of HCP, emphasizing the need to enhance the competence and confidence of CNAs and NAs, particularly due to insufficient RN coverage. The participants stressed the need for training NAs to help them recognize changes in residents that should be reported to CNAs or RNs. One dyad noted the diverse backgrounds of NAs, which range from medical or nursing students to individuals studying not health-related fields like economics, making it difficult to address their varying skill levels. Additionally, the extent of advanced assessments conducted by CNAs was described as varying widely depending on individual competency, with experienced CNAs demonstrating strong observational and assessment skills.

Several dyads emphasized the importance of implementing the intervention nursing home-wide, where RNs who are not permanently assigned to the respective unit often hold the overall nursing care responsibility for the residents.

I think it is a very useful tool for establishing a common language and a consistent approach to assessment. This makes it easier when on-call staff need to be involved, as it becomes simpler to track what has already been done. Often, you find yourself in situations where you don't know what to do, which is a challenge when you're an on-call RN. (.) This should be implemented throughout the entire nursing home (Dyad 6).

Participants provided specific suggestions for further developing the intervention content, such as a module on measures and treatment, a dedicated structure for pain reporting at the RN level, pocket cards and tools for assessing pain risk in new residents. One dyad highlighted that “receiving” expressions of pain is a subjective experience for HCP, emphasizing the need to address not only the resident's experience of pain but also HCP's experience and interpretation. Nursing home units were described to operate differently, requiring the intervention to be adapted to the various models they follow. The dyads underscored that it must be “woven” into existing practices.

#### Implementation strategies

The dyads proposed context-specific implementation strategies, highlighting varied preferences for organization of training. They generally favored e-learning, particularly when combined with in-person meetings, underscoring the importance of practical training and opportunities for reflection and discussion. E-learning was seen as flexible and able to reach a broad audience, while in-person training was described as motivational and a positive break from the routine. Two dyads highlighted the benefits of one-on-one or smaller group training focused on specific patient cases, which helped build confidence in observations and reporting.

Combining theory with reflection is the most valuable approach and should be directly related to practice. I often use real events that have occurred, because it helps everyone understand that this is real, something we've experienced, something they've felt firsthand (Dyad 5)

Participants stressed the need for intervention content to be accessible to all staff and noted that using a flowchart requires training, as it introduces a new way of thinking.

Several dyads noted the use of “change champions” with a strong understanding of the intervention and high motivation. Some suggested the practice development nurses as natural change champions. Others stressed the importance of including representatives from various HCP groups and suggested the formation of a project group with participants from multiple units, led by a nursing home representative. This was also expected to facilitate long-term implementation, in addition to integrating training into the onboarding process for new employees. Establishing the initiative at a higher organizational level, such as nursing home management or the municipal level, was seen as critical for justifying financial priorities.

### Potential moderators

Moderators are factors that positively or negatively influence the intervention's mechanisms. Conversely, mechanisms can also impact moderators; for example, intrinsic motivation and competence are identified as moderators but also described as something the intervention can enhance. This section categorizes the identified moderators as individual or organizational, in line with the predefined analytical framework.

#### Individual

The participants described inner moderators such as personal qualities, confidence in role, motivation, competence and resistance to change. They underscored the importance of HCP personal suitability in pain management, noting that a flowchart alone is insufficient. HCP confidence in their role was highlighted as essential for pain management. Those directly working with the residents, along with their motivation, are crucial for interpreting and understanding residents' expressions and ensuring appropriate pain management as outlined in the flowchart.

Staff motivation was described as a moderator, but the intervention itself was seen as a potential source of motivation, as its goal is to improve resident care. Involvement in the development and planning of the intervention, as in this project, was described as inherently motivating by the participants. Previous positive implementation experiences were noted as a moderator, while resistance to change was seen as inevitable by several dyads. One dyad described the importance of accepting that some individuals initially will be negative but often shift their perspectives as the process progresses.

Not always, but most people who are negative about something new in the beginning tend to shift their perspectives over time (dyad 7).

#### Organizational

The findings describe organizational moderators related to staffing levels and organization of RNs and physicians, structures and priorities of competence development, leadership, ward culture and electronic resources.

Participants underscored the critical importance of an organizational structure facilitating thorough and time-intensive assessments for RNs. The nursing homes had varying practices in relation to the physicians. Some had experienced geriatricians skilled in dementia care and familiar with care models, while others highlighted the need to train junior doctors lacking such expertise. All dyads emphasized the importance of including physicians in the intervention.

The nursing homes differed in their organization of training, and training facilities such as simulation rooms. Several dyads noted challenges in organizing in-person training for NAs, who often work evening shifts or have other jobs or studies during the daytime. When present during daytime, NAs are often not prioritized for training, as they are needed on the ward while permanent staff attend sessions.

All dyads highlighted leadership commitment. Managers must allocate time and clearly communicate that the intervention will be implemented in practice. This was also linked to the need to set clear expectations for staff and guide them by emphasizing that the use of it is expected, desired and required. Leadership was identified as closely linked to ward culture. Dyads highlighted the importance of fostering a culture where staff are confident in their roles and value their contributions to pain management. Building confidence, particularly among NAs and CNAs, and strengthening the professional environment were deemed essential. Well-designed forms, while important, were noted to be only one aspect of optimal pain management.

It is equally important to feel confident in your role and to foster a culture and practice that supports this. This foundation is essential for providing effective pain relief. Without it, the information won't be fully utilized. You can have all the forms of assessments you want, but if the staff are not confident in their roles, it won't work—that foundation must be in place. (Dyad 4).

The dyads emphasized integrating the intervention into digital systems instead of relying on paper-based documentation. Limited availability of electronic resources to attend e-learning on the units was noted, and mobile-friendly e-learning and easy access to digital resources were considered essential.

### Potential outcomes

The intervention targets HCP and their behavior in pain management for people with dementia, with outcomes related to HCP described first in this section. Resident-related outcomes, focusing on residents with dementia as the ultimate beneficiaries, are presented second. The outcomes in this section are prospective, reflecting participants’ expectation of the intervention's potential impact.

#### Healthcare personnel

The dyads indicated that the intervention is expected to enhance competence and confidence. Improved knowledge enables CNAs and NAs to observe and assess within their role and function. The intervention is also expected to foster support among CNAs and reduce missed observations. The intervention has the potential to activate the “team” around the resident, including significant others, and enhance interdisciplinary collaboration among HCP. Accurate documentation, such as RNs using a standardized structure for pain reports, is expected to support physicians in making more informed decisions. The intervention may also improve collaboration with on-call RNs by providing a structured approach to “control questions” (e.g., “What have you observed? Why do you think this?”). Clearer role definition is anticipated to increase CNA involvement in pain management, while physicians may gain a better understanding of RN assessments through insight into ward pain management processes.

The intervention was expected to enhance HCP motivation by ensuring that all staff feel their observations are valued. Working in a person-centered manner is described as increasing job satisfaction, fostering intrinsic motivation, and providing a sense of reward by enabling staff to contribute to effective measures. Additionally, it is anticipated to promote a greater sense of mastery.

Increased awareness of pain management is anticipated, fostering a mindset where staff consistently focus on the fundamental aspects of pain management and actively identify signs of pain. This heightened awareness is anticipated to extend to other residents, improving overall recognition of pain. The guidance provided by the intervention on how to approach and think about pain is described as essential, with one participant stating, “It helps us think”. All dyads highlighted that this intervention could offer a more systematic and consistent approach to pain management, creating a stronger foundation for assessment. It ensures that everyone understands the process, even if they are not directly involved in all aspects. The intervention is expected to bring greater depth to evaluations and encourage further investigations if initial measures are ineffective. It is anticipated to support a more comprehensive and holistic approach to pain management. However, the dyads acknowledged that challenges would remain:

Even with such assessments, it is still a person who must interpret them. These tools can help us become more aware, potentially reducing variability in subjective assessment—because they guide and raise awareness about how we should evaluate and think. However, the assessment remains subjective, as it is not objective data. We will never know with 100% certainty (Dyad 4).

Unintended outcomes of the intervention may include “false pain alarms”, as there is a considerable behavior that may not be pain-related, or overmedication caused by continuously increasing doses due to the assumption that behavior is pain-related.

#### Resident

The participants described how these HCP-related outcomes can subsequently lead to resident outcomes, emphasizing that they could help ensure that people with dementia are acknowledged in their expressions of pain. Improved interaction with the resident may contribute to pain alleviation, partly by helping the resident feel seen and acknowledged. The dyads expected the intervention to contribute to optimized pain management by supporting residents in coping with pain and ensuring the appropriate use of analgesics. They emphasized that the residents could experience an improved daily life if pain is alleviated through proper management. At unit level, the intervention may lead to a reduction in behavioral and psychological symptoms in dementia, which also includes fewer general pain expressions that are difficult to interpret.

Although prevention has not been the primary focus of the intervention, several dyads highlighted that it has the potential to contribute to pain prevention.

Providing attention, conducting examinations, and offering time and connection can potentially alleviate pain by ensuring that individuals feel acknowledged, taken seriously, and seen (Dyad 1).

The dyads described the “ultimate goal” as achieving improved service quality for people with dementia, where each resident experiences either pain relief or the ability to live with their pain and thereby enhancing their quality of life.

## Discussion

In this study a prototype of an intervention targeting healthcare personnel managing pain in people with dementia in nursing homes was presented to stakeholders. The aim was to describe stakeholders’ perspectives of the prototype regarding intervention content and relevance, and implementation strategy. The results are described in relation to core constructs of the intervention: activities, potential moderators and potential outcomes (38). This knowledge is essential for further development and adaptation of a sustainable intervention. This discussion section highlights two aspects: first, the results, and second, this study's approach to intervention development.

### Results discussion

Caspar et al. (43) identifies three key factors required for achieving changes in care practices: predisposing, enabling, and reinforcing. They highlight the importance of obtaining preliminary input from stakeholders about their needs. The approach taken in this study, as well as in prior studies within the Palliative care to Older People with dementia project, directly addresses this. Predisposing factors include effective communication and dissemination of information. Our findings highlight the need to adapt the intervention content, such as the language used in the flowchart, to align with existing practices. Adaptations will vary between nursing homes depending on their established care models and organizational structures. Such flexibility is likely essential for effective implementation.

Enabling factors refer to conditions and resources that allow staff to apply their newly acquired skills, such as administrative support (43). As our results describe, enabling factors can include support materials like the flowchart, pocket card and documentation structures, but also already established factors such as patient safety boards or implemented models for person-centered care. The participants further highlighted the importance of creating an environment where knowledge and flowchart are prioritized and expected to be used, ensuring they are integrated into practice. A clear and structured approach, such as the flowchart, was identified as another enabling factor, as it provides concrete guidance on applying knowledge.

Reinforcing factors involve mechanisms to ensure the sustained implementation of new skills or practices (43). These may include recognition, a sense of achievement, supervision, and structures to maintain the intervention. Participants in this study described the potential “reward” of seeing the intervention benefit residents and improve daily practice. However, Dahl et al. (44) found that staff often struggled to apply established knowledge consistently, due to residents' unpredictable behavior and responses. Reinforcing factors proposed by participants included training new employees, ongoing education, and establishing teams with “change champions” who continuously work on pain management. Participants also mentioned factors like motivation through rewards for completing training modules, hands-on supervision by practice development nurses or “change champions”, and participation in training or reflection groups as motivators and enjoyable breaks from daily routine. Caspar et al. (43) emphasizes that HCP are more likely to adopt practice changes when predisposing, enabling and reinforcing factors are in place. For a flexible intervention strategy to succeed, it might be essential to ensure that all three are consistently incorporated.

The moderators described in this study are consistent with findings from other studies on moderators in interventions targeting person-centered care and/or education of HCP (45–48). Nakrem et al. (48) problematize the challenges of implementing change in nursing home practices, attributing this to a lack of understanding the complexity of nursing home care and limited awareness of the relationship between individual nursing practices and organizational factors. Kormelinck et al. (47) suggests that while some moderators are generic in nature, several “setting-specific” factors also influence implementation. They emphasize the importance of focusing on modifiable moderators, as factors perceived as obstacles in one organization may not be seen as a barrier in another. This highlights the need to account for local conditions and specific moderators by developing tailored implementation plans (47). Conducting this study made it possible to identify context-specific moderators, recognizing that moderators can vary between different contexts.

This study gives insight into the participants' perspectives on potential outcomes of the intervention. This not only supports the planning of evaluation but also contributes to the creation of shared goals, which is identified as an enabling factor for successful implementation (43). Muller-Schoof et al. (29) describe *evaluation* as an integral part of the implementation strategy. By gathering feedback on the testing and implementation process from various stakeholders, evaluation helps refine strategies and identify moderators. An ongoing feedback loop is described to not only enhance the effectiveness of implementation strategies but also fostering a culture of learning and adaptation (29). Similarly, Bourbonnais et al. (49) adjusted their implementation strategies throughout the implementation process based on barriers identified along the way and suggestions from care staff. They highlight that, while it was impossible to eliminate all barriers, adapting the strategies allowed for successful implementation.

Overall, the findings of this study align with the notion that dissemination and education should be combined with strategies involving (a) social influence, (b) collaboration, (c) regulation, and (d) evaluation, as described by Muller-Schoof et al. (29). They stress that implementation strategies must be multifaceted, combined, and context-specific to address the complexities of nursing home settings.

*Social influence* is a strategy that motivates HCPs by involving key individuals who demonstrate and explain new practices. Participants suggested project groups or “change champions” representing diverse HCP roles. Such role models might create a safe space for discussion and learning, helping teams understand and adopt new practices. Observing trusted peers implement changes successfully and discussing them collectively fosters openness to new approaches (29). Champions can overcome implementation barriers independently (45) and are most effective when they exhibit qualities like flexibility, commitment, leadership, eagerness to learn, and enthusiasm for improving dementia care (46).

The participants described how being involved in the development of the intervention served as a motivating factor. *Collaboration* includes a co-creative partnership where stakeholders actively influence both processes and outcomes, such as plans, advice, or products. This approach enhances the understanding and application of research findings in nursing home practice through dialogue and mutual learning (29). Although *mandating laws or regulations* was not a focus in our findings, the participants highlighted the value of anchoring interventions at a higher management or political level to justify resource allocation. Clear demands and expectations from nursing home management were also deemed vital. However, the participants stressed that HCP must view the intervention as beneficial to residents; external mandates perceived as adding administrative burden without improving resident care may reduce motivation and increase resistance among staff.

### Approach to intervention development

In addition to addressing interventions targeting HCP's pain management for people with dementia in nursing homes, this study demonstrates an applied approach to intervention development which incorporates stakeholders' perspectives. While this method of intervention development offers distinct strengths, it also presents certain challenges.

Including several stakeholder groups, rather than solely service providers, has been shown to highlight critical areas and structures for consideration in intervention development (50). CNAs and RNs participated in a previous study in this project, contributing the ideas that form the foundation of the prototype (17). However, given the emphasis on the structural and organizational changes required for sustainable practice change (45), both practice development nurses and first-line managers are crucial stakeholders. While they are service providers, they bring dual expertise: clinical care for people with dementia and organizational knowledge, including familiarity with moderators and experience implementing changes and building staff competence in nursing homes. This aligns with the importance of considering context when selecting stakeholders.

The second decision to discuss in this study is the degree of stakeholder involvement. Co-design approaches are a broad and variable concept but include a commitment to power-sharing with stakeholders in decisions (51). While co-design, in which stakeholders act as equal co-researchers, is often considered a gold-standard, it can be time-intensive and challenging, particularly in resource-constrained contexts such as nursing homes (52). In this project, no funds have been allocated to the nursing homes to backfill or otherwise free stakeholders' clinical time. Consequently, given the current pressure on clinical service, we cannot reasonably expect stakeholders to leave their clinical duties to take on full responsibilities as co-researchers. However, they can still be meaningfully included in the development process without assuming such responsibilities. This study demonstrates how stakeholders can be engaged in a pragmatic approach to intervention design, ensuring their input is valued without placing undue burden on them.

Rousseau et al. (53) identified six modes of intervention design, varying in the degree of stakeholder involvement. They distinguish between «informed» and «negotiated» design, noting that some interventions shifted between modes during development. The approach in this study aligns most closely with what they call «informed design» mode. Stakeholder engagement functions as an information source, evaluated alongside other forms of knowledge. In this study, stakeholders are consulted to validate the emerging design and contribute ideas, but key decisions are made by the development team.

Thereto, Rousseau et al. (53) argue that intervention design extends beyond creating the intervention itself to include two critical aspects: (a) ideation—generating ideas about the interventions form, components, and delivery methods, and (b) decision-making—determining which ideas to include or discard. They suggest that incorporating design thinking into co-creation processes can enable stakeholders to participate meaningfully. This study used an interview guide that focuses on specific intervention components. The interview guide clarified the type of input required at this stage of the design process, providing a structured frame that directed stakeholders' contributions. This approach has the potential to reduce perception of symbolic involvement by ensuring that their ideas can be meaningfully incorporated into the intervention.

### Refinement and further development of the intervention

The next step involves refining the intervention content and planning the intervention strategy based on the results of this study, followed by a small-scale evaluation. This refinement includes adjustments such as adapting module content, for example by adding a module on pain measures, developing pocket cards and a structured documentation template for RNs, and incorporating flexible elements that allow nursing homes to adapt the flowchart to their specific organizational structures. The pedagogical approach could combine e-learning with touchpoints for training, discussion, and reflection. Insights into potential moderators allow for their inclusion in the evaluation process, as well as targeted efforts to address moderators both prior to and during implementation. Participants' perspectives on potential outcomes provide valuable guidance for planning the evaluation. For example, HCP confidence emerged as a more significant outcome in pain management than initially anticipated, and the role of NAs is now considered more central, highlighting the importance of including them in the evaluation process. Participants expressed strong support for the intervention. The feasibility of the intervention will be tested by HCP, enabling a nuanced understanding of its outcomes through a combination of quantitative and qualitative data. These findings will, in turn, inform further revisions to the program theory.

## Conclusion

This study presents an applied approach to collaborating with stakeholders in the development of an intervention targeting pain management for people with dementia living in nursing homes. The intervention prototype was deemed relevant, and the results describe participants' perspectives on activities, moderators and potential outcomes of the intervention. This provides essential knowledge for further refinement of the intervention and planning the future evaluation.

## Data Availability

The datasets presented in this article are not readily available because the raw data that support the findings of this study are not available due to confidentiality and ethical restrictions. Requests to access the datasets should be directed to caroline.kreppen.overen@ldh.no.
